# Exploring Antibacterial Activity of Fish Protein Hydrolysate In Vitro Against *Vibrio* Strains and Disease Resistance to *V. harveyi* in Turbot (*Scophthalmus maximus*)

**DOI:** 10.1155/anu/3446155

**Published:** 2025-01-28

**Authors:** Yuliang Wei, Lu Wang, Yanlu Li, Qiang Ma, Mengqing Liang, Houguo Xu

**Affiliations:** ^1^State Key Laboratory of Mariculture Biobreeding and Sustainable Goods, Yellow Sea Fisheries Research Institute, Chinese Academy of Fishery Sciences, Qingdao, Shandong 266071, China; ^2^Laboratory for Marine Fisheries Science and Food Production Processes, Qingdao Marine Science and Technology Center, Qingdao, Shandong 266237, China

**Keywords:** antibacterial activity, fish protein hydrolysate, immune response, intestinal microbiota, *Vibrio*

## Abstract

This study was to investigate the in vitro antimicrobial activity of fish protein hydrolysate (FPH) against *Vibrio harveyi*, *V. anguillarum*, and *V. scophthalmi*, as well as the nonspecific immunity, intestinal microbiota, and disease resistance to *V. harveyi* in turbot. FPH was prepared from Pollock. The antibacterial activity of FPH was measured by the agar well diffusion, turbidometric assay, and plate count. The feeding trial was performed to study the effect of FPH on the resistance against *V. harveyi* in turbot after feeding three diets containing a high level of fish meal (FM), a high level of soybean meal (SM), and 100 g/kg FPH. Agar well diffusion showed the clearest inhibition zone of FPH was observed against *V. harveyi*, followed by *V. scophthalmi*. The bacterial growth curve and plate count showed a slight antibacterial effect of FPH against *V. anguillarum*. Results of the feeding trial showed that FPH enhanced antioxidant and immune responses before *V. harveyi* challenge as modulating immunoglobulin M (IgM), catalase (CAT), and myeloperoxidase (MPO) activities in serum, as well as the number of goblet cells in the intestine. Meanwhile, the expression of some pro-inflammatory cytokines (interleukin-1*β* [*il-1β*], *il-6*, and *il-8*) was downregulated in the FPH group after the *V. harveyi* challenge. Survival probability in the FPH group increased after challenging to *V. harveyi* based on the Kaplan–Meier analysis. Results of intestinal microbiota showed the relative abundance of *Vibrio* in the SM group was the highest, followed by the FPH and control groups. Similarly, the relative abundance of distal intestinal *V. harveyi* was significantly reduced in the FPH group by analyzing the *vhhp2* gene. In conclusion, the present FPH against *Vibrio* strains was species-specific, with stronger antibacterial activity to *V. harveyi*. Dietary FPH enhanced the nonspecific immunity and antibacterial activity of turbot, increasing the resistance to *V. harveyi*.

## 1. Introduction

The fish processing industry generates a large amount of fish byproducts while meeting human demand for aquatic animal food [1]. According to the production of wild and farmed fish, the global fish byproducts are estimated to reach 22.2 million tons per year [2], which will result in the disposal problems and environmental pollution. Since fish byproducts can be considered a valuable source of fish protein, its treatment in an environmentally friendly and sustainable manner has driven research into fish protein hydrolysate (FPH) [3]. Using exogenous enzymatic hydrolysis to produce FPH was an effective method to recycle and efficiently utilize fish byproducts. FPH is a product containing free amino acids and peptides by enzymatically hydrolyzing native protein from fish byproducts [4]. In terms of the nutritional property of FPH, it not only enhances fish growth as the protein source substitute to fish meal (FM) but also improves utilization of some low-quality ingredient sources, such as poultry byproduct meal [5], soy meal concentrate [6], and lupin meal/fermented lupin meal [7], by compensating for their nutritional shortcomings [8].

In addition, antibacterial peptides are found to be produced during the hydrolysis process of FPH. For instance, Aissaoui et al. [9] reported that an antibacterial peptide with the sequence FPIGMGHGSRPA was purified from *Scorpaena notata* viscera protein hydrolysate using a neutral protease; Offret et al. [10] discovered a glyceraldehyde−3-phosphate dehydrogenase-related antibacterial peptide (KVEIVAINDPFIDL) from Atlantic mackerel (*Scomber scombrus*) hydrolysate that exhibited antibacterial activity against *Listeria* strains. In the food nutrition aspect, numerous studies focused on the antibacterial activity of FPH against many food-spoiling bacteria, such as *Escherichia coli*, *Salmonella* spp., *L. monocytogenes*, *Staphylococcus aureus*, and used its antibacterial properties as the natural ingredient for food production industry [11, 12]. In the aquaculture aspect, FPH was considered as the immunomodulatory to stimulate the nonspecific immune defense system of fish [13]. Decreased infection rates and increased survival rates were recorded in some fish species after challenging to pathogenic bacteria [14–16]. Antibacterial peptides from fish have the potential for using them as therapeutic agents in aquaculture due to their antimicrobial activities and immunomodulatory functions. However, antibacterial peptides often exhibited species-specific antibacterial activities, which have proven to be specific inhibitory abilities against different bacteria [17, 18]. Nowadays, there are few reports on whether FPH has a direct antibacterial effect on pathogenic bacteria commonly found in aquaculture. Therefore, an evidence of the antibacterial activity of FPH against aquaculture-associated pathogens is needed to facilitate its application in aquafeeds.

In our previous study with turbot (*Scophthalmus maximus*), the analysis of intestinal microbiota using 16S rRNA sequencing revealed that a moderate amount of FPH reduced the abundance of the distal intestinal *Vibrio* at the genus level [19]. It implied that FPH may affect the abundance of *Vibrio* in the intestine. In turbot culture, *Vibrio* spp. was the most important pathogen causing the outbreak of bacterial diseases [20]. Pathogenic *Vibrio* strains have been identified, including *V. harveyi* [21], *V. anguillarum* [22], and *V. scophthalmi* [23]. In this study, we first analyzed the antibacterial activity of FPH against the above three *Vibrio* strains in vitro. Then, one of the strains was chosen, which FPH had the strongest inhibitory activity against it. After that, this study further investigated the effect of FPH on growth performance, nonspecific immune response, intestinal microbiota, and disease resistance of turbot before and after being challenged with the selected *Vibrio* strain.

## 2. Materials and Methods

### 2.1. Antibacterial Activity of FPH

#### 2.1.1. Production of FPH

The hydrolysis method reported by Zheng et al. [24] had been used to obtain FPH. Byproducts of Pollock (*Theragra chalcogramma*) were minced using a B-400 mixer (Büchi, Switzerland). Then, 100 g of sample was mixed with 100 mL of distilled water and heated at 90°C for 10 min to inactivate the endogenous enzymes. The reaction of enzymatic hydrolysis was conducted with two enzymes of Alcalase (Novozymes, Bagsvaerd, Denmark) and Flavourzyme (Novozymes). Fish byproducts were hydrolyzed with Alcalase and Flavourzyme with enzyme-substrate ratios of 1:1:100 (v:w:w). The initial pH value was adjusted to 8.5–9.0, and then the pH was not adjusted again throughout the hydrolysis process. The temperature was maintained at 55°C for 3 h. At the end of the reaction, the mixture of enzyme and substrate was heated gradually from 55 to 90°C and then kept at 90°C for 20 min to ensure enzyme inactivation. The mixture was centrifuged at 3000 *g* for 3 min. The supernatant was the liquid FPH, and the powdered sample was then obtained by the processes of rotary evaporation and freeze–drying. FPH was stored at −20°C for subsequent experiments.

#### 2.1.2. Preparation of Microbiological Culture Medias With FPH

The composition of microbiological culture medias (tryptic soy broth, TSB) is provided in Table 1. The proteinaceous materials (tryptone and soy peptone) in the TSB medium were replaced by FPH at the levels of 0%, 25%, 50%, 75%, and 100% to keep the iso-nitrogen. The TSB media without FPH (0%) was used as the control treatment.

#### 2.1.3. Bacteria and Antibacterial Assays

*V. harveyi*, *V. anguillarum*, and *V. scophthalmi* were obtained from the Division of Maricultural Organism Disease Control and Molecular Pathology, the Yellow Sea Fisheries Research Institute, and the Chinese Academy of Fishery Sciences. Isolated *V. harveyi*, *V. anguillarum*, and *V. scophthalmi* bacteria cultures were inoculated in TSB agar plates and incubated at 37°C for 24 h. The cultured isolate was inoculated in 5 mL TSB medium with 120 rpm/min at 37°C for 5–6 h, and the optical density (OD, *λ* = 600) value was adjusted to be 0.5. Isolated *V. harveyi*, *V. anguillarum*, and *V. scophthalmi* were sent for 16S rRNA sequencing, and then nucleotide sequences were used to run a blast at NCBI to validate the three strains of *Vibrio*.

The antibacterial activity was first measured using the agar well diffusion method descripted by Aqil et al. [25] with some modifications. The inoculum (150 μL) with *V. harveyi*, *V. anguillarum*, or *V. scophthalmi* was spread evenly on TSB agar plates. About 6 mm diameter wells were punched in the agar medium (4 mm depth) and filled with 20 µL of 500, 1000, and 1500 mg/mL FPH, 20–100 μg/mL the antibiotic (florfenicol) (the positive treatment), and 15 mg/mL NaCl (the negative treatment) based on our preliminary experiments. The plates were incubated at 37°C for 24 h in an aerobic environment.

Turbidometric assay was used to measure the antibacterial activity according to the method reported by Raghupathi et al. [26] with some modifications. The activated bacteria were prepared from isolated colonies of agar streak plates and then were inoculating at 37°C in 5 mL TSB until the OD value at 600 nm was 1.0 based on the eliminated background (TSB). And then, different bacteria were diluted 1/500 in the TSB medium supplemented with graded levels of FPH (Table 1). The bacterial growth curves were obtained by measuring the OD at 600 nm. The OD values of bacteria were monitored every 2 for 24 h, except *V. scophthalmi*, which was monitored for 30 h.

The concentration of the target bacteria was measured by the plate count method to evaluate the antibacterial activity [27, 28]. The activated bacteria from isolated colonies of agar streak plates were diluted in 2 mL physiological saline. And then, 200 μL of bacterial cell suspensions were added to 3 mL test medium with graded levels of FPH (Table 1). The liquid inoculums were incubated at 37°C until one of the OD values (*λ* = 600) from five test medium reached 1.0. Subsequently, the bacteria were gradually diluted to 10^−5^, 10^−6^, and 10^−7^ dilutions (about 1000, 100, and 10 CFUs). About 200 μL aliquots of each dilution were placed to agar plates and incubated at 37 °C for 24 h. Colonies (30–300 CFUs) were counted manually to calculate the concentration of the target bacteria.

### 2.2. Experimental Diets, Experimental Fish, and Feeding Trial

Three isonitrogen and isolipid diets were formulated at ~500 g/kg crude protein and ~100 g/kg crude lipid. The control diet contained a high level of FM (480 g/kg) and a low level of plant protein mixture (100 g/kg soybean meal [SM], 50 g/kg wheat gluten, and 60 g/kg corn gluten meal). According to the studies with turbot reported by Bonaldo et al. [29] and Gu et al. [30], 100 g/kg FM in the control diet was replaced by 150 g/kg SM to serve as the SM diet. In addition, 100 g/kg FPH (the FPH diet) based on our previous studies [19, 31] was supplemented to the SM diet at the expense of 100 g/kg FM. The formulation and proximate composition of experimental diets are presented in Table 2. To prepare the diets, all the ingredients except FPH were crushed and sieved through 0.18 mm sieve, and then the ingredients were mixed well step by step according to the experimental formulation. FPH was dissolved with water, added into the ingredients, and mixed well. Pellets with a diameter of about 3 mm were made by a laboratory scale pelleter, and the diets were stored at −20°C before use.

The experimental fish were turbot with an initial body weight of about 47 g, purchased from Shandong Kehe Ocean High Technology Co. Ltd. (Weihai, China) and transported to Huanghai Aquaculture Co. Ltd. (Yantai, China) for the feeding trial. Fish were temporarily reared for 3 weeks using a commercial feed to acclimatize to the culture conditions. Then, fish were starved for 24 h and randomly assigned to nine tanks (0.7 m × 0.7 m × 0.4 m). Three diets were assigned to the tanks (*n* = 3), with 30 fish in each tank. Each tank of fish was divided into three batches, and fish were individually selected in each batch, with a total of 10 fish selected and weighed and then randomly placed into the tank. Fish were fed to apparent satiation at 7:00 and 18:30 in a flow-through culture system with individual air stones. During the 44-day culture period, the water temperature was 17–19°C, the salinity was 30, the pH was about 7.8, and the dissolved oxygen content was 6–7 mg/L.

### 2.3. Sample Collection

At the start of the feeding trial, 10 turbot were randomly taken for proximate analysis of the body and stored at −20°C. At the end of the feeding trial, fish were starved for 24 h and then weighed and counted to calculate the parameters related to growth performance. Six fish were randomly taken from each tank and anesthetized with MS-222 at 30 mg/L. Blood samples were collected from the caudal vasculature to 1.5-mL tubes using heparinized needles and then were allowed to clot at 4°C for 4 h and centrifuged at 4000 *g* for 10 min to obtain the serum. After blood sampling, four turbot were dissected and randomly taken from each tank to collect head kidney and intestine (proximal, middle, and distal) tissues based on our previous study [32]. Parts of intestinal tissues from the four fish were also collected and fixed in Bouin's solution for histological processing. The remaining two fish from each tank were randomly selected and stored at −20°C for body composition analysis [33].

### 2.4. *V. harveyi* Challenge

The above-mentioned *V. harveyi* was used in the current bacterial challenge experiment. *V. harveyi* was inoculated on 2216E medium and incubated at 37°C for 24 h for the recovery of the bacteria, followed by expanded incubation on 2216E plates at 37°C for 24 h. The strains were diluted with physiological saline to 1 × 10^8^ CFU/mL in the suspension based on our preliminary LD50 experiments. The remaining turbot, after sampling, were injected intraperitoneally with a dose of 200 μL per fish. Fish were checked at 12-h intervals after injection, and the number of dead fish was recorded. During the period of bacterial challenge, fish did not consume the experimental diet because of the injection stress. Sampling was initiated when the first mortality was observed at 24 h following injection. The specific sampling procedure was the same as the sampling procedure described in Section 2.3, except that intestinal tissues for histological processing and whole fish were not collected.

### 2.5. Serum Biochemical, Antioxidant, and Immunological Parameters

The detections of albumin (ALB), total protein (TP), total superoxide dismutase (T-SOD), catalase (CAT), glutathione peroxidase (GSH-PX), malondialdehyde (MDA), myeloperoxidase (MPO), glutamic oxalic aminotransferase (GOP), glutamic pyruvic aminotransferase (GPT), lysozyme (LZM), and total nitric oxide synthase (T-NOS) in the serum had been performed using their respective commercial kits (ALB assay kit, cat#A028-2-1; the TP assay kit, cat#A045-4-2; T-SOD assay kit, cat#A001-1-2; CAT assay kit, cat#A007-1-1; GSH-PX assay kit, cat#A005-1-2; MDA assay kit, cat#A003-1-2; MPO assay kit, cat#A044-1-1; aspartate aminotransferase assay kit, cat#C010-2-1; alanine aminotransferase assay kit, cat#C009-2-1; LZM assay kit, cat#A050-1-1; T-NOS assay kit, cat#A014-2-2) following the manufacturer's protocols. The levels of immunoglobulin M (IgM) and Complement 3 (C3) in the serum were measured using two commercial kits (IgM assay kit, cat#H109-1-2; C3 assay kit, cat#H186-1-2) with enzyme-linked immunosorbent assay (ELISA). All commercial kits used in this study were provided by NanJing JianCheng Bioengineering Institute (Nanjing, China).

### 2.6. Gene Expression Analyses

Gene expression analyses were performed based on our previous study [34]. Briefly, total RNA from the head kidney, proximal intestine, and middle intestine were extracted using AG RNAex Pro Reagent (cat#AG21102, Accurate, Changsha, China), and cDNA templates were synthesized using Evo M-MLV RT Mix Kit (cat#AG11705, Accurate, Changsha, China). The amplification efficiencies of the primers for the target and reference genes were kept ranging from 95% to 105% using a standard curve with four-fold dilution. Relative expression of target genes was analyzed using the 2^−∆∆*Ct*^ method after quantitative real-time-polymerase chain reaction (qRT-PCR). *β*-Actin and ribosomal protein L19 (rpl19) were served as the reference genes (Table 3). The qRT-PCR program included an initial denaturation at 95°C for 30 s, followed by 40 cycles (95°C, 5 s; 57°C, 30 s; 72°C, 30 s) and end with a melt curve analysis provided by a Roche LightCycler 96 system (Roche, Switzerland). The reaction was carried out in 10 μL of total volumes, including 1 μL of cDNA template, 5 μL of SYBR Green Pro Taq HS Premix II (cat#AG11702, Accurate, Changsha, China), 0.3 μL of forward primer, 0.3 μL of reverse primer, and 3.4 μL of sterilized water.

### 2.7. DNA Extraction and 16S rRNA Sequencing

The samples of the middle and distal intestines were sent for DNA extraction and 16S rRNA sequencing at the Shanghai Majorbio Bio-pharm Technology Co. Ltd. (Shanghai, China). Briefly, intestinal tissues were subjected to DNA extraction using a MagAtrract PowerSoil Pro DNA Kit (cat# 4898129, Qiagen, Hilden, Germany). The bacterial 16S rRNA genes were amplified using the premiers of 27F (5′-AGRGTTYGATYMTGGCTCAG−3′) and 1492R (5′-RGYTACCTTGTTACGACTT−3′). The PCR products were purified using the AMPure PB beads (Pacific Biosciences, CA, USA) and quantified with Qubit 4.0 (Thermo Fisher Scientific, MA, USA). The DNA library was constructed using the SMRTbell prep kit 3.0 (Pacific Biosciences, CA, USA) and then sequenced on the Pacbio Sequel IIe System (Pacific Biosciences, CA, USA). High-fidelity (HiFi) reads were generated from the subreads by the CCS mode of SMRT-Link v11.0. HiFi reads were identified based on the barcode, and the sequences of 1000−1800bp were retained. UPARSE 7.1 (http://drive5.com/uparse/, version 7.1) was used to obtain operational taxonomic units (OTUs) based on the optimized-HiFi reads with 97% sequence similarity. RDP classifier (http://rdp.cme.msu.edu/, version 2.11) was used to annotate OTU species taxonomies against the Silva 16S rRNA gene database (Silva v138), with a confidence threshold of 70%, and the community composition of each sample was counted at different species classification levels. Alpha diversity indices, including Sobs, Shannon, Simpson, Ace, Chao1, and Pielou-e, were calculated with Mothur v1.30.1 (http://www.mothur.org/wiki/Calculators).

### 2.8. Intestinal Morphology

Fixed samples from middle and distal intestine tissues were processed for histological analysis based on the procedure reported in our previous study [19]. Samples were dehydrated in ethanol dilutions and embedded in paraffin to obtain intestinal transversal sections. Paraffin-embedded tissues were cut at 5 µm thickness and then stained with hematoxylin solution and eosin dye. The morphology of the intestine was photographed with a Pannoramic MIDI scanner (3DHISTECH Ltd., Hungary). The investigation of histology was focused on villus height, wall thickness, and goblet cell and processed by the ImageJ.

### 2.9. The qRT-PCR Assay in Detecting *V. harveyi* of Middle and Distal Intestine After the Challenge

The *vhhp*2 gene (NCBI GenBank: FJ025787.1) was used as a specific target gene of *V. harveyi* for the qRT-PCR assay. The primers (Vh-F: 5′- GTGGTGGCGAACTGGATGTAA −3′; Vh-R: 5′- GAGTATTCGTTGCCGAGTAAAGC -3′) with 151-bp PCR product had been used to detect *V. harveyi* [35].

The designed primers of *vhhp*2 gene were validated, and a standard curve was established according to the precious method with some modifications [36, 37]. The *V. harveyi* strain was activated, and the genomic DNA was extracted using the TIANamp Bacteria DNA Kit (cat# DP302, Tiangen, Beijing, China). The concentration of the genomic DNA was diluted to 100 ng/μL with sterile water. The bacterial genomic DNA (100 ng/μL) was re-diluted to 4^−1^, 4^−2^, 4^−3^, 4^−4^, 4^−5^, 4^−6^, 4^−7^, 4^−8^, 4^−9^, and 4^−10^ dilutions, which were used as templates for qRT-PCR based on the primers of *vhhp*2 gene. The reaction system was 5 μL SYBR Green Pro Taq HS Premix II, 0.25 μL Vh-F (10 μmol/L), 0.25 μL Vh-R (10 μmol/L), 0.5 μL DNA template, and 4 μL sterilized water. The qRT-PCR was programed as follows: 95°C for 30 s and 40 cycles (95°C, 5 s; 60°C, 30 s; 72°C, 30 s). After that, genomic DNA was extracted from the middle and distal intestine of turbot using the TIANamp Marine Animals DNA Kit (cat# DP324, Tiangen, Beijing, China). The qRT-PCR was carried out using Vh-F/Vh-R mentioned above as primers and 100 ng/μL genomic DNA as templates. The reaction system was as follows: 5 μL SYBR Green Pro Taq HS Premix II, 0.25 μL Vh-F (10 μmol/L), 0.25 μL Vh-R (10 μmol/L), 2 μL DNA template, and 2.5 μL sterilized water. The qRT-PCR conditions was 95°C for 30 s and 45 cycles (95°C, 5 s; 60°C, 30 s; 72°C, 30 s; 72°C, 30 s).

### 2.10. Statistical Analysis

The data, except for the survival analysis after the *V. harveyi* challenge and the relative abundance of OTUs at the phylum and genus levels, were presented as mean values ± standard error. These data were analyzed by a one-way analysis of variance (ANOVA) test (SPSS 16.0, IBM Company, Chicago, IL, USA). And then, a Tukey-HSD test was used for multiple comparisons when significant differences (*p* < 0.05) were detected. The survival analysis after *V. harveyi* challenge was subjected to Kaplan–Meier analysis with a log-rank (SPSS 16.0). In addition, the parameters (serum biochemical, antioxidant, and immunological parameters, the gene expression of immune-related inflammatory cytokines in the proximate intestine, middle intestine, and head kidney, as well as the *α*-diversity index of the distal intestinal microbiota) before and after *V. harveyi* challenge were compared using independent sample *T*-test (SPSS 16.0).

## 3. Results

### 3.1. Antibacterial Activity of FPH

**Figure 1:**
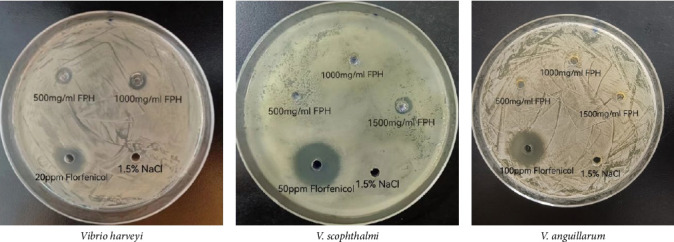
Antimicrobial activity of fish protein hydrolysate (FPH) against *Vibrio harveyi* (A), *V. scophthalmi* (B), and *V. anguillarum* (C). The experiments were repeated at least three times, and representative results were presented in the figures.

Figure 1 shows the antibacterial activity of FPH against *V. harveyi*, *V. scophthalmi*, and *V. anguillarum*. Although the inhibition zones were very narrow, they were observed against *V. harveyi* at 500 and 1000 mg/mL of FPH and against *V. scophthalmi* at 1500 mg/mL of FPH. In addition, FPH did not generate the inhibition zone for *V. anguillarum* at 500, 1000, and 1500 mg/mL of FPH.

**Figure 2:**
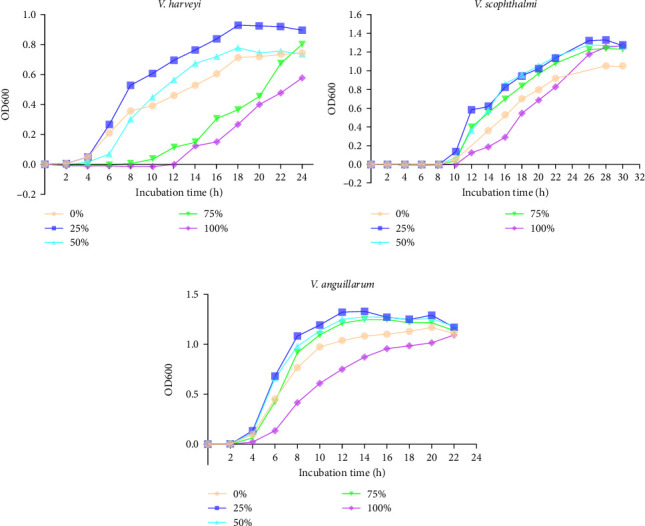
Inhibitory effect of graded levels of fish protein hydrolysates (FPH) on bacterial [(A) *V. harveyi*; (B) *V. scophthalmi*; and (C) *V. anguillarum*] growth. The *x*-axis indicates the incubation time (h), and the *y*-axis indicates the optical density (OD) measured at 600 nm. The experiments were repeated at least three times, and the average OD was presented in the figures.

Figure 2 shows the growth curve of *V. harveyi*, *V. scophthalmi*, and *V. anguillarum* in the presence of graded levels of FPH. FPH at the level of 100% inhibited the growth of *V. harveyi*, *V. scophthalmi*, and *V. anguillarum* compared to that at the level of 0%. The inhibitory effect of the growth of *V. harveyi* was observed in the presence of FPH at the level of 50% and 75% (incubation time 2–8 h) compared to the 0% FPH. In addition, no inhibitory effect of 25% FPH was detected in any of the bacterial growth.

Table 4 shows bacterial counts of *V. harveyi*, *V. scophthalmi*, and *V. anguillarum* in microbiological culture medias with graded levels of FPH. *V. harveyi* count in the level of 25% FPH was significantly higher than that of the level of 0% FPH (*p* < 0.05), while no *V. harveyi* were detected in culture medias of 50%, 75%, and 100% FPH. *V. scophthalmi* count in the level of 50% FPH was significantly higher than that of 100% FPH (*p* < 0.05) but significantly lower than that of 0% and 25% FPH (*p* < 0.05). *V. anguillarum* count decreased significantly (*p* < 0.05) only when the replacement level of FPH reached 50% and 100% in the culture medias compared with the control group (0% FPH).

### 3.2. The Growth and Feed Utilization

Table 5 shows the growth and feed utilization in turbot before *V. harveyi* challenge. There were no significant differences in final body weight, weight gain, specific growth rate, feed intake, feed conversion ratio, protein efficiency ratio, and protein productive value among the dietary treatments (*p* > 0.05). However, final body weight, weight gain, and specific growth rate in the fish fed the diet containing a high level of SM had a decreasing trend (*p* > 0.05) compared to the FPH diet with the same level of SM. The survival rate was 84.4% in the SM group, which was more than 10% lower than that in the control and FPH groups, although there was no significant difference between treatments (*p* > 0.05).

### 3.3. Histology of Middle and Distal Intestine

**Figure 3:**
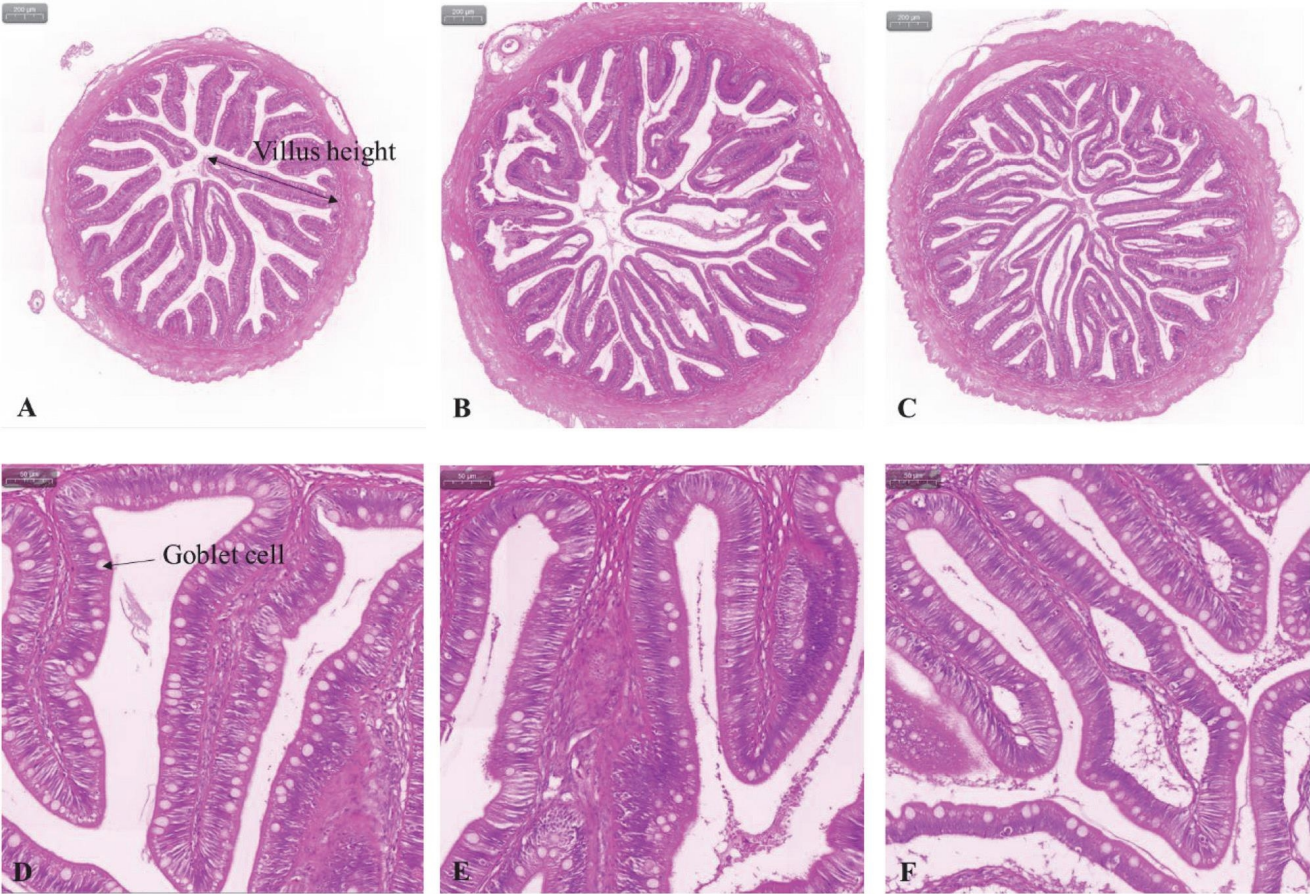
Example images of middle intestinal histology in turbot before *V. harveyi* challenge: (A, B, C) represent the Control, SM, and FPH group at a 40x magnification, respectively; (D, E, F) represent the Control, SM, and FPH group at a 200x magnification, respectively. FPH, fish protein hydrolysate; SM, soybean meal.

**Figure 4:**
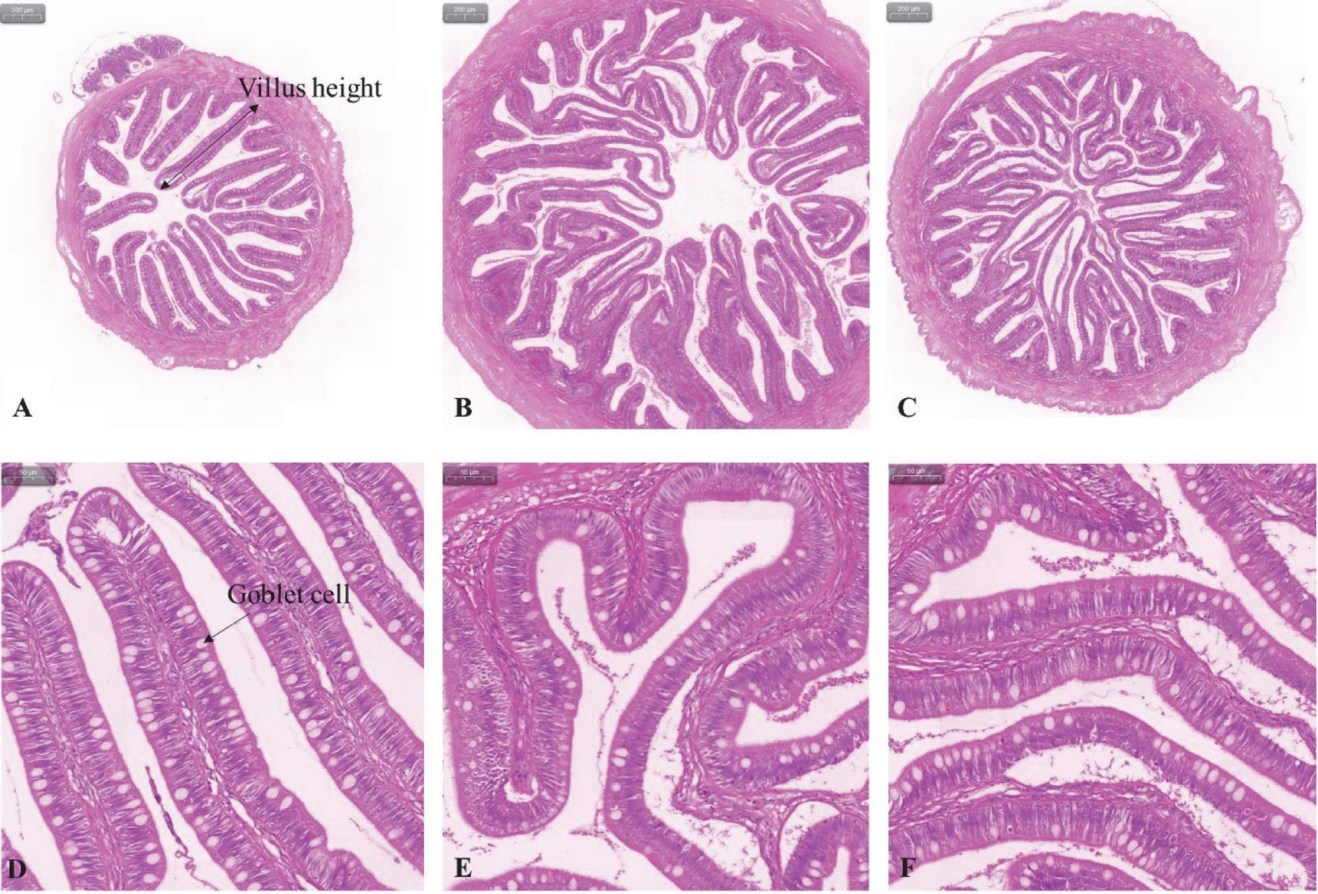
Example images of distal intestinal histology in turbot before *V. harveyi* challenge: (A, B, C) represent the Control, SM, and FPH group at a 40x magnification, respectively; (D, E, F) represent the Control, SM, and FPH group at a 200x magnification, respectively. FPH, fish protein hydrolysate; SM, soybean meal.

Table 6, Figures 3 and 4 show the histology of the middle and distal intestine in turbot before *V. harveyi* challenge. The number of goblet cells in the middle intestine was the highest in the FPH group at 1319.83, which was 27.79% and 43.69% higher than that in the control and SM group, respectively, although there was no significant difference (*p* > 0.05) between them. In the distal intestine, the proportion of villus height (> 1000 mm) in the SM group was significantly higher (*p* < 0.05) than that of the control and FPH groups. Similar to the results of the middle intestine, the number of goblet cells in the distal intestine was the highest in the FPH group at 1111.33, which was 26.84% and 20.95% higher (*p* > 0.05) than that of the control and SM groups, respectively.

### 3.4. Serum Biochemical, Antioxidant, and Immunological Parameters

Table 7 shows the biochemical, antioxidant, and immunological parameters of serum in turbot before or after *V. harveyi* challenge. The activities of CAT and MPO, as well as the level of IgM in serum, were significantly (*p* < 0.05) affected by dietary treatments before *V. harveyi* challenge. The activities of serum CAT and MPO in the FPH group were significantly lower (*p* < 0.05) compared with the control and SM groups, while fish fed the FPH diet exhibited higher (*p* < 0.05) level of serum IgM.

After *V. harveyi* challenge, the activity of LZM, as well as the levels of ALB, TP, and MDA, were significantly (*p* < 0.05) affected by dietary treatments. Fish fed the SM diet showed the lowest levels (*p* < 0.05) of serum ALB, TP, and MDA. In addition, the activity of serum LZM in the control group was significantly lower (*p* < 0.05) than that of the SM and FPH groups.

Serum biochemical, antioxidant, and immunological parameters of all treatments were compared before and after *V. harveyi* challenge (Supporting Information 2: Figure S1). The levels or activities of biochemical (TP), antioxidant (T-SOD, GSH-PX, and MDA), and immunological (NOS) parameters in all the treatments were significantly higher (*p* < 0.05) before the challenge than those after the challenge. The level of Alb from the SM and FPH groups, the activity of CAT from the SM group, and the level of IgM from the FPH group were significantly higher (*p* < 0.05) before the challenge compared with those after the challenge. In addition, the activities of MPO from the control and FPH groups, as well as GPT from all the groups, significantly increased (*p* < 0.05) after the challenge.

### 3.5. The Gene Expression of Immune-Related Inflammatory Cytokines

**Figure 5:**
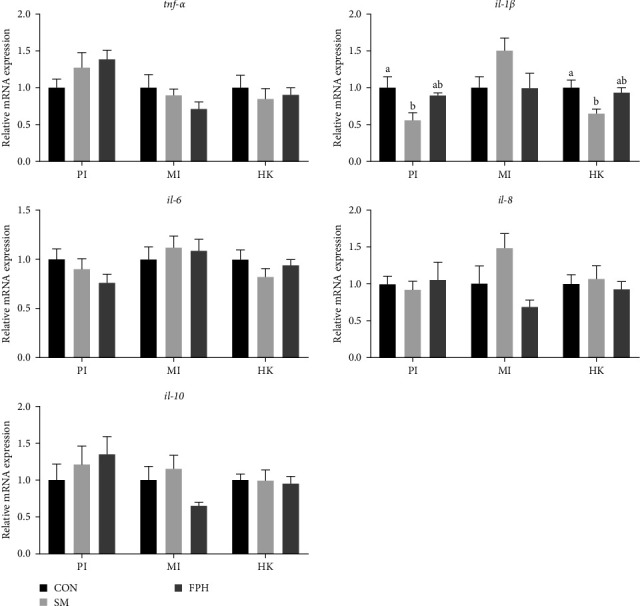
The gene expression of immune-related inflammatory cytokines in the proximate intestine (PI), middle intestine (MI), and head kidney (HK) of turbot before *V. harveyi* challenge. *il-1β*, interleukin-1*β*; *tnf-α*, tumor necrosis factor *α*. Values are means ± standard error of three replicate tanks. Bars with different letters are significantly different (*p*  < 0.05) according to Tukeys' multiple comparison test.

Figure 5 shows the gene expression of immune-related inflammatory cytokines in the proximate intestine, middle intestine, and head kidney of turbot before *V. harveyi* challenge. Among the four pro-inflammatory cytokines (*il-1β*, interleukin-1*β*; *tnf-α*, tumor necrosis factor *α*; *il-6*; *il-8*), only the expression of *il-1β* was significantly higher (*p* < 0.05) in the proximate intestine of the control group than that of the SM group, but there was no significant difference (*p* > 0.05) between the control and the FPH group. The expression of *il-1β* in the head kidney among all the treatments showed a similar trend compared with it in the proximate intestine. The anti-inflammatory factors (*il-10*) in the intestine, middle intestine, and head kidney did not exhibit a significant difference (*p* > 0.05) between dietary treatments.

**Figure 6:**
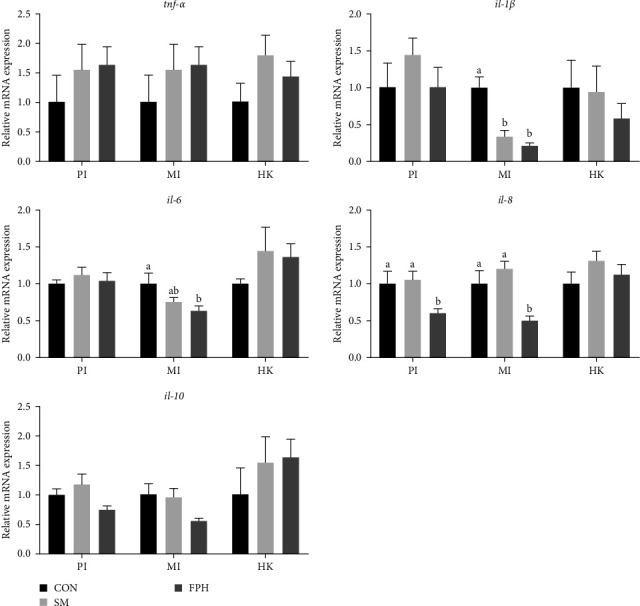
The gene expression of immune-related inflammatory cytokines in the proximate intestine (PI), middle intestine (MI), and head kidney (HK) of turbot after *V. harveyi* challenge. *il-1β*, interleukin-1*β; tnf-α*, tumor necrosis factor *α*. Values are means ± standard error of three replicate tanks. Bars with different letters are significantly different (*p* < 0.05) according to Tukeys' multiple comparison test.

Figure 6 shows the gene expression of immune-related inflammatory cytokines after *V. harveyi* challenge. The expression of middle intestine *il-1β* was significantly downregulated (*p* < 0.05) in fish fed the SM and FPH diets compared with the control diet. The expression of *il-6* in the middle intestine and *il-8* in the proximate and middle intestines exhibited a similar trend between treatments, where the lowest mRNA level (*p* < 0.05) was observed in fish fed the FPH diet.

The gene expression of immune-related inflammatory cytokines in the proximate intestine, middle intestine, and head kidney were compared before and after *V. harveyi* challenge (Supporting Information 2: Figures S2–S4). The expression of the proximate intestine, middle intestine, and head kidney *il-1β* and *il-8* in all the groups was significantly upregulated (*p* < 0.05) after the challenge than that before the challenge. For the expression of *il-10*, significant upregulation (*p* < 0.05) after the challenge was observed in the head kidney from all the groups and in the proximate intestine from the control and SM groups, while significant downregulation (*p* < 0.05) was observed in the middle intestine from the FPH group. In addition, the expression of *tnf-α* after the challenge was significantly lower in the middle intestine from all the groups compared with that before the challenge.

### 3.6. Survival Analysis and Relative Concentration of *V. harveyi* in the Intestine

**Figure 7:**
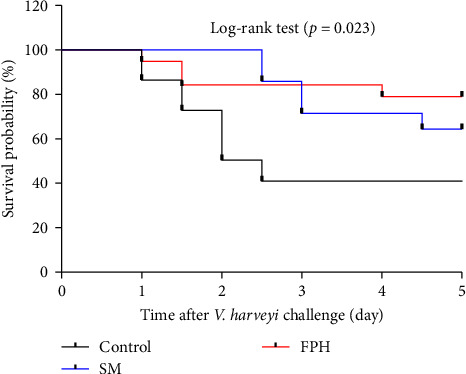
Survival analysis of turbot after *V. harveyi* challenge. Kaplan–Meier survival curve was used to estimate the overall survival rate between treatments. Daily fish deaths were recorded after *V. harveyi* challenge with *n* = 30 for each treatment.

Figure 7 shows the survival analysis of turbot after *V. harveyi* challenge by using the Kaplan–Meier survival curve. The first fish mortality was recorded in the control and FPH groups at 24 h after *V. harveyi* challenge. Survival probability in fish fed the control diet was lower than 50% at 2 days after *V. harveyi* challenge. In addition, from the 2 days of *V. harveyi* challenge to the 5 days, the mortality rate observed in the FPH group was much lower than that in the control and SM groups. At the 5 days of *V. harveyi* challenge, the highest of survival probability was observed in the FPH diet. Based on the log-rank test, the *p* value was 0.023, which indicated that survival curves were significantly affected by dietary treatments.

**Figure 8:**
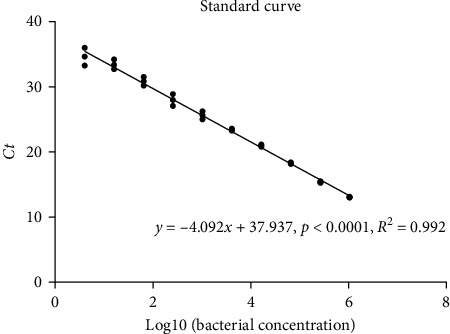
Standard curve of the relative concentration of *V. harveyi*. The *x*-axis indicates *Ct* value provided by qRT-PCR based on the primers of *vhhp2* gene, and the *y*-axis indicates concentrations of *V. harveyi* genomic DNA for log10 transformation. *Ct* values are detected for three replicate samples (*n* = 3). qRT-PCR, quantitative real-time-polymerase chain reaction.

Figure 8 shows the standard curve of relative concentration of *V. harveyi*. The concentration of *V. harveyi* was transformed by the log 10 transformation, which was decreased linearly with *Ct* value of the *vhhp*2 gene using the qRT-PCR assay. It confirmed that the qRT-PCR assay for the *vhhp*2 gene could be used to assess the relative abundance of *V. harveyi*.  Table 8 shows *Ct* value of the *vhhp2* gene in the middle and distal intestine after *V. harveyi* challenge based on this method. The lowest *Ct* value of the *vhhp2* gene in the middle intestine was observed in the control group, while there was no significant difference (*p* > 0.05) between dietary treatments. In the distal intestine, the *Ct* value of the *vhhp2* gene in the FPH group significantly increased (*p* < 0.05), followed by the SM and control groups. It indicated that the relative concentration of *V. harveyi* in the distal intestine was FPH < SM < Control.

### 3.7. Microbiota of the Distal Intestine

Table 9 shows an *α*-diversity index of the distal intestinal microbiota of turbot before or after *V. harveyi* challenge. Regardless of *V. harveyi* challenge, all *α*- diversity parameters (Sobs, Shannon, Simpson, Ace, Chao1, and Pielou-e) at the phylum and genus levels were no significant differences (*p* > 0.05) between treatments. In addition, *α*-diversity parameters of the distal intestinal microbiota in all the treatments were compared before and after the challenge, but no significant differences (*p* > 0.05) were observed at the phylum and genus levels (Figures S5 and S6).

**Figure 9:**
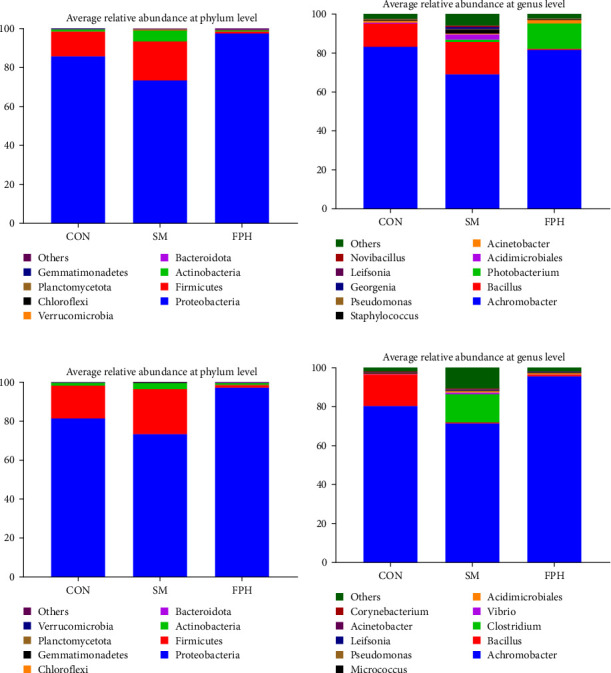
Average relative abundance of the distal intestinal microbiota of turbot. (A) The average relative abundance (*n* = 3) at the phylum level before *V. harveyi* challenge; (B) the average relative abundance (*n* = 3) at the genus level before *V. harveyi* challenge; (C) the average relative abundance (*n* = 3) at the phylum level after *V. harveyi* challenge; and (D) the average relative abundance (*n* = 3) at the genus level after *V. harveyi* challenge.

Figure 9 shows the average relative abundance of the distal intestinal microbiota. The most dominance of distal intestinal microbiota at the phylum level were *Proteobacteria* and *Firmicutes* (Supporting Information 1: Tables S1, S2), which were not affected by experimental diets or by *V. harveyi* challenge. At the genus level, the relative abundance of *Achromobacter* was the highest among all the groups both before and after *V. harveyi* challenge. Before *V. harveyi* challenge, the *Bacillus* genus was the core microbiota in the control and SM groups with a relative abundance of 11.83% and 16.63%, respectively, while the *Photobacterium* genus was the core microbiota in the FPH group with a relative abundance of 13.06%. However, after *V. harveyi* challenge, *Bacillus* (16.33%) was still the core microbiota of the control group, and the relative abundance of *Achromobacter* in the FPH group increased to 95.5%, but no relative abundance exceeded 1% at the level of the remaining genera. Additionally, no *Vibrio* genus was found in the top 10 genera in all treatment groups before *V. harveyi* challenge, but the relative abundance of *Vibrio* genus increased significantly to the fourth dominant microbiota after *V. harveyi* challenge. The relative abundance of *Vibrio* genus in the SM group was the highest, followed by the FPH and control groups.

## 4. Discussion

FPH used in this study was made from byproducts of Pollock by enzymatic hydrolysis (Alcalase and Flavourzyme), which had been repeatedly verified for its growth-promoting effect on turbot [19, 31, 32]. Wang et al. [38] reported that growth performance and intestinal health of turbot would be deteriorated by high SM inclusion in diets. In this study, based on previous studies with turbot [29, 30], the level of SM in the SM and FPH diets was increased to 250 g/kg by replacing FM with SM. However, no statistically significant differences in growth and feed utilization were found between treatment groups. One possible explanation was that this study focused on investigating the immune and antibacterial effects of FPH; therefore, about 47 g of turbot with relatively higher initial body weight (more convenient sampling for intestinal microbiota analysis) were selected, and the period of the feeding trial was only 44 days (the experimental diets were almost exhausted at 44 days). It may result in the growth-promoting effects of FPH being masked to some extent due to the low weight gain causing by the short rearing period. However, the trend of positive effect of FPH on growth was still observed; 250 g/kg SM in diet reduced weight gain by 3.73% compared to the control (FM), while the value of weight gain in fish fed diet with FPH restored and exceed fish fed the control diet. It indicated that, even under the conditions of the present experiments, it was still partially to demonstrate that FPH indeed improved the utilization of SM and promoted fish growth.

In addition, the high-level inclusion of SM in diets induced intestinal inflammation in turbot [30]. Typical signs of enteritis in the intestine of turbot were a shortening of villus height, upregulated gene expression of pro-inflammatory cytokines, and downregulated gene expression of anti-inflammatory cytokines [38, 39]. In this study, these signs of enteritis in the intestine were not observed in the villus height and gene expression of inflammatory cytokines before *V. harveyi* challenge. The severity of enteritis caused by SM differed between fish species [40–42]. Gu et al. [30] reported that turbot had a higher tolerance to SM-induced inflammatory response, where the level of SM was ranging from 260 to 540 g/kg. It indicated that 250 g/kg SM in diets was likely the onset of the reaction in the intestine, which resulted in no marked enteritis before *V. harveyi* challenge. However, based on the comparison of the expression levels of these inflammatory cytokines before and after the challenge, it was found that the expression levels of *il-1β*, *tnf-α*, *il-8*, and *il-10* were significantly increased or decreased in all treatment groups. *V. harveyi* has been shown to induce the upregulated expression of inflammatory cytokines via the p38 MAPK (mitogen-activated protein kinases) and nuclear factor-kappa B (NF-*κ*B) signal pathways [43]. It was supported by this study, where *V. harveyi* challenge induced the inflammatory response in the proximate and middle intestine or head kidney of turbot. Furthermore, after *V. harveyi* challenge, the expression of some pro-inflammatory cytokines (*il-1β*, *il-6*, and *il-8*) was downregulated in fish fed the FPH diet compared to fish fed the SM or FM diet. Therefore, the current results suggested that FPH inclusion in diets may reduce the risk of inflammation occurring in the intestine due to *V. harveyi* infection [44].

Dietary nutrients are considered to play an important role in influencing the immunity and disease resistance of fish [45]. Plasma immune-related and antioxidant parameters are widely used as indicators in assessing the immunity and health status of fish [46, 47]. It was partly supported in this study, where the comparison of serum biochemical, antioxidant, and immunological parameters before and after *V. harveyi* challenge exhibited that some parameters (TP, T-SOD, GSH-PX, MDA, NOS, and GPT) of all treatment groups significantly increased or decreased. However, before the challenges, the sampling was done for 24 h of fish starvation, whereas after the challenge, the sampling was done for 48 h of fish starvation because the fish did not consume the experimental diets due to the injection stress. Therefore, the changes in those parameters before and after the challenges may not be caused only by *V. harveyi* challenge. In addition, FPH can function as an immune-stimulant to stimulate nonspecific immunity against pathogenic bacteria [48]. FPH supplementation enhanced nonspecific immune responses, such as the LZM activity as well as the levels of complement and IgM, which were recorded in barramundi (*Lates calcarifer*) [49], Japanese sea bass (*Lateolabrax japonicas*) [50], olive flounder (*Paralichthys olivaceus*) [51], larger yellow croaker (*Pseudosciaena crocea* R.) [52], red sea bream (*Pagrus major*) [53]. In this study, before challenging with *V. harveyi*, serum IgM level significantly increased in fish fed the diet supplemented with FPH, and the level of C3 increased by 13.9% and 32.1% (no statistical differences) compared with the control and SM group, respectively. In addition, the present results showed that LZM activity in both the FPH and SM groups was significantly higher than that in the control group after *V. harveyi* challenge, but it was not possible to determine whether the increase was caused by the substitution of FM by SM or FPH supplementation. MPO plays a key role in activated neutrophils during the immune response, which is function as the oxygen-dependent bactericidal activity. However, excessive release of MPO will be harmful for intact host tissues [54]. The activity of MPO in turbot fed the SM diet was significantly higher compared to the control diet before *V. harveyi* challenge, which indicated that a high level of SM in the diet resulted in the massive release of MPO. However, the FPH fed to the turbot reduced the possible damaging effects of SM on tissues caused by elevated MPO activity. This study also found the number of goblet cells in fish fed the FPH diet tended to be elevated in the middle and distal intestine, and similar results were observed in olive flounder [51] and Pompano (*Trachinotus blochii*) [15]. Goblet cells can exhibit improvement in innate immune responses of the intestine due to the secretion of mucus, antibacterial proteins, chemokines, and cytokines [55]. Taken together, nonspecific immune responses may be facilitated when FPH was supplemented to the diet of turbot. In addition, some studies have confirmed that FPH could improve the antioxidant capacity of some fish species, such as largemouth bass (*Micropterus salmoides*) [56] and barramundi [57]. The main reason was that antioxidant peptides were present in the FPH, which could directly donate hydrogen atoms to reactive oxygen species, deactivate molecular oxygen, scavenge free radicals, and chelate metal ions [4]. These antioxidant properties of FPH, in turn, reduced the activity of some antioxidant enzymes, such as superoxide dismutase [16]. Similar results were observed in this study, where the activity of CAT significantly decreased in fish fed the FPH diet before *V. harveyi* challenge. Therefore, FPH enhancement of antioxidant capacity may also been observed in turbot.

The study of the antibacterial activity of FPH in vitro has previously been mainly in the field of food science. The reason is that FPH contains antimicrobial peptides, which can damage the integrity of the cell membrane by forming pores or by blocking the membrane ion gradients [58]. Furthermore, some antimicrobial peptides cause the death of bacteria by disrupting cellular metabolism instead of lysing the cell membrane [59, 60]. Therefore, FPH has been demonstrated its antibacterial effect against food-borne pathogenic bacteria, including Gram-positive (*S. typhimurium* and *E. coli*) and Gram-negative bacteria (*L. innocua* and *L. monocytogenes*) [12]. The antibacterial property of FPH is dependent on the raw material of hydrolysis, the enzyme type, the degree of hydrolysis, and molecular weight distribution [11, 12, 61]. In addition, the antibacterial activity of FPH is species-specific, for example, the inhibitory effect of FPH derived from Nile tilapia (*Oreochromis niloticus*) byproducts had a high sensitivity to *S. typhimurium* and *L. monocytogenes*, but a low sensitivity to *E. coli* [11]. In the present study, the result of the inhibition zone based on the agar well diffusion method showed that FPH prepared from Pollock byproducts in vitro exhibited the highest antibacterial activity against *V. harveyi*, followed by *V. scophthalmi*. Moreover, according to the bacterial growth and bacterial counts, FPH used in this study seemed to have a slightly antibacterial effect on *V. anguillarum*. These results indicated that FPH was bacteriostatic against pathogenic *Vibrio* strains from marine fish but was species-specific as the case with food-associated pathogens.

As for fish nutrition, the main focus is on evaluating the resistance of FPH in vivo against pathogenic bacteria after infection. For example, 20 g/kg FPH supplementation in diets increased the resistance to *Streptococcus iniae* in Nile tilapia [62] and to *Aeromonas hydrophila* in Pabda (*Ompok pabda*) [63]; Siddik et al. [49, 64] claimed that adding 61 g/kg improved survival rates in barramundi against *V. harvei* and *S. iniae* infection. In turbot, although there were no reports of FPH against pathogenic challenge, previous analysis of intestinal microbiota found that FPH reduced the abundance of *Vibrio* at the genus level [19]. However, in the current study, *Vibrio* was not observed among 10 dominant genera of intestinal microbiota before *V. harveyi challenge*. It may be due to the different rearing sites for the two experiments; the previous one was conducted at the Yantai Tianyuan Aquatic Product Co. Ltd., while the present one was conducted at Huanghai Aquaculture Co. Ltd. The composition of intestinal core microbiota may be altered by environmental rearing conditions [65], which had been reported in Atlantic salmon (*Salmo salar* L.) [66]. However, 24 h after intraperitoneal injection of *V. harveyi* to turbot, the abundance of intestinal *Vibrio* at the genus level significantly increased by analyzing 16S rRNA sequencing, demonstrating an altered composition of distal intestinal microbiota, especially the abundance of *Vibrio*. Meanwhile, the relative abundance of intestinal *V. harveyi*, as determined based on the qRT-PCR assay for the *vhhp2* gene, was significantly reduced in the distal intestine of a turbot-fed diet containing FPH. These results were consistent with the abundance of distal intestinal *Vibrio* at the genus level and survival curves after *V. harveyi* challenge, which proved the inhibitory effect of FPH on *V. harveyi* in vivo. However, considering that after intraperitoneal injection of *V. harvey*, turbot refused to eat the experimental diets due to the injection stress, increased resistance to *V. harvey* herein mainly relied on FPH to improve nonspecific immune response. Similar results were found in the study with red seabream (*Pagrus major*), where increased the resistance to *Edwardsiella tarda* was by improving the innate immunity [67].

## 5. Conclusion

The present study revealed that the antimicrobial effect of FPH prepared from byproducts of Pollock exhibited species specificity for *Vibrio* strains from marine fish. Compared to *V. scophthalmi* and *V. anguillarum*, FPH showed stronger antibacterial activity against *V. harveyi*. Adding 100 g/kg FPH enhanced the nonspecific immunity of turbot by regulating a few antioxidant and immunological parameters of serum, as well as intestinal goblet cell, the abundance of distal intestinal *Vibrio* at the genus level, and pro-inflammatory cytokines, resulting in increasing resistance capacity to *V. harveyi*.

## Figures and Tables

**Table 1:** Microbiological culture medias with graded levels of fish protein hydrolysate (FPH)^a^.

Ingredient	0%	25%	50%	75%	100%
Tryptone (g)	17.00	12.75	8.5	4.25	0.00
Soy peptone (g)	3.00	2.25	1.5	0.75	0.00
FPH (g)	0.00	6.07	12.14	18.21	24.28
Glucose (g)	2.5	2.5	2.5	2.5	2.5
K_2_HPO_4_·3H_2_O (g)	2.5	2.5	2.5	2.5	2.5
NaCl (g)	10	10	10	10	10
Protein content (%)	18.64	18.64	18.64	18.64	18.64

^a^Tryptone and soy peptone were replaced by 0%, 25%, 50%, 75%, and 100% FPH of total protein.

**Table 2:** Formulation and proximate composition of the experimental diets (g/kg dry matter).

Ingredients	Control	SM	FPH
Fish meal	480	380	280
SM	100	250	250
Wheat gluten	50	50	50
Corn gluten meal	60	60	60
FPH	0	0	100
Wheat meal	201	145	139
Fish oil	63	69	75
Soybean lecithin	15	15	15
Mineral premix^a^	5	5	5
Vitamin premix^b^	10	10	10
Ca (H_2_PO_4_)_2_	10	10	10
Choline chloride	4	4	4
Vitamin C	2	2	2
Proximate composition			
Moisture	50.5	44.6	78.4
Crude protein	512.0	513.0	528.4
Crude lipid	97.4	96.9	102.0
Ash	101.4	98.1	92.0

Abbreviations: FPH, fish protein hydrolysate; SM, soybean meal.

^a^Mineral premix (g/kg premix): FeSO_4_ · H_2_O, 112.7 g; ZnSO_4_ · H_2_O, 45.2 g; MnSO_4_ · H_2_O, 9.3 g; CuSO_4_ · 5H_2_O, 3.7 g; CoCl_2_ · 6H_2_O, 0.4 g; Na_2_SeO_3_, 0.1 g; Ca (IO_3_)_2_, 0.3 g.

^b^Vitamin premix (IU or g/kg premix): Vitamin A acetate, 1,140,000 IU; Vitamin D3, 180,000 IU; DL-*α*-tocopherol acetate, 7.6 g; Menadione, 1.2 g; Thiamin nitrate, 0.93 g; Riboflavin, 1.35 g; Pyridoxine hydrochloride, 1.10 g; Cyanocobalamine, 0.0075 g; D-Calcium pantothenate, 4.5 g; Nicotinamide, 6.75 g; Folic acid, 0.465 g; D, biotin; 0.0475 g; Inositol, 10 g.

**Table 3:** Sequence of primers for quantitative real-time-polymerase chain reaction (qRT-PCR).

Gene	Primer sequence (5′−3′)	Product length	Gene Bank^a^
*il-1β*	F-CTACCTGTCGTGCCAACA R-TAGAACAGAAATCGCACCAT	117	XM_035640817.2
*il-6*	F-AATCACCCACGGCTGCATTA R-TTGCATTGTCCCAGAGCCAT	92	XM_035621201.2
*il-8*	F-GGCAGACCCCTTGAAGAATA R-TGGTGAACCCTTCCCATTAT	138	XM_035638413.2
*tnf-α*	F-TAGGCTTCAACGCGTCTCTG R-CTGGAACACTGCACCCAGAT	122	FJ654645.1
*il-10*	F-AACGGGCTGATGCTCTAACC R-GAGCGTAAGGCTGGCTAGTT	108	XM_035632547.2
*β-actin*	F-CCAAAGCCAACAGGGAGAA R-AGAGGCATACAGGGACAGCACA	101	AY008305.1
*rpl19*	F-GCACATGTACCACAGCCTCT R-GGCCTCCTTCGTCTTAGAGC	157	XM_047328333.1

Abbreviations: *il-1β*, interleukin-1*β*; qRT-PCR, quantitative real-time-polymerase chain reaction; *rpl19*, ribosomal protein L19; *tnf-α*, tumor necrosis factor *α*.

^a^NCBI GenBank accession no.

**Table 4:** Bacterial counts of *V. harveyi*, *V. scophthalmi*, and *V. anguillarum* in microbiological culture medias with graded levels of fish protein hydrolysate (FPH) (CFU/mL).

Microorganism	0%	25%	50%	75%	100%
*V. harveyi*	[(3.0 ± 0.50) × 10^8^]^b^	[(6.1 ± 0.5) × 10^8^]^a^	—	—	—
*V. scophthalmi*	[(6.4 ± 0.7) × 10^8^]^a^	[(4.5 ± 0.3) × 10^8^]^a^	[(1.3 ± 0.0) × 10^8^]^b^	[(1.3 ± 0.1) × 10^8^]^bc^	[(9.1 ± 0.8) × 10^7^]^c^
*V. anguillarum*	[(1.8 ± 0.2) × 10^8^]^a^	[(2.2 ± 0.3) × 10^8^]^a^	[(2.1 ± 0.2) × 10^8^]^a^	[(1.1 ± 0.1) × 10^8^]^b^	[(2.9 ± 0.2) × 10^7^]^b^

*Note:* Values are means and standard error of three replicate treatments. Values in the same row followed by different superscript letters are significantly different (*p*  < 0.05). The data of *V. harveyi* was subjected to *T*-test. Other data were log10 transformed and then subjected to one-way analysis of variance (ANOVA) with Tukey's multiple comparison test.

**Table 5:** The growth and feed utilization of turbot.

Item	Control	SM	FPH
Initial body weight (g)	47.00 ± 0.58	47.44 ± 0.99	48.67 ± 1.15
Final body weight (g)	77.36 ± 3.73	76.33 ± 2.12	80.33 ± 3.05
Survival rate^a^ (%)	95.56 ± 1.11	84.44 ± 4.44	95.55 ± 2.22
Weight gain^b^ (%)	64.61 ± 7.88	60.88 ± 3.20	65.01 ± 4.06
Specific growth rate^c^ (%/day)	1.13 ± 0.11	1.08 ± 0.05	1.14 ± 0.05
Feed intake^d^ (%/day)	1.59 ± 0.10	1.70 ± 0.02	1.80 ± 0.08
Feed conversion ratio^e^	1.57 ± 0.18	1.60 ± 0.05	1.63 ± 0.11
Protein efficiency ratio^f^	1.34 ± 0.06	1.21 ± 0.04	1.17 ± 0.09
Protein productive value^g^ (%)	23.42 ± 3.04	21.51 ± 3.04	18.70 ± 0.32

*Note:* Values are means and standard error of three replicate tanks. Values in the same row followed by different superscript letters are significantly different (*p*  < 0.05) according to Tukeys' multiple comparison test.

Abbreviations: FPH, fish protein hydrolysate; SM, soybean meal.

^a^Survival rate (%) = 100 × (final fish number/initial fish number).

^b^Weight gain (%) = 100 × (final body weight − initial body weight)/initial body weight.

^c^Specific growth rate (%/day) = 100 × [ln (final body weight) − ln (initial body weight)]/feeding days.

^d^Feed intake (%/day) = 100 × total feed intake/[feeding days × (initial body weight + final body weight)/2].

^e^Feed conversion ratio = dry feed intake/body weight gain.

^f^Protein efficiency ratio = (final body weight − initial body weight)/dietary protein intake.

^g^Protein productive value (%) = 100 × (crude protein content of final body − crude protein content of initial body)/dietary protein intake.

**Table 6:** Histology of middle and distal intestine in turbot before *V. harveyi* challenge.

Item	Control	SM	FPH
Middle intestine			
The distribution (%) of villus height			
0–100 (μm)	12.80 ± 5.11	22.42 ± 4.61	12.00 ± 2.34
101–500 (μm)	53.67 ± 4.79	40.65 ± 2.48	45.21 ± 4.83
501–1000 (μm)	31.45 ± 4.74	29.70 ± 2.12	38.87 ± 1.73
>1000 (μm)	2.08 ± 0.86	7.23 ± 2.63	3.92 ± 1.47
The number of goblet cell	1032.83 ± 102.57	918.50 ± 116.03	1319.83 ± 97.71
Distal intestine			
The distribution (%) of villus height			
0–100 (μm)	20.66 ± 5.19	10.61 ± 1.57	10.03 ± 4.05
101–500 (μm)	47.22 ± 6.39	40.63 ± 2.39	58.13 ± 8.28
501–1000 (μm)	32.12 ± 1.24	36.11 ± 4.99	26.76 ± 8.00
>1000 (μm)	0^b^	12.35 ± 1.90^a^	5.08 ± 2.56^b^
The number of goblet cell	876.17 ± 137.86	918.83 ± 110.83	1111.33 ± 176.10

*Note:* Values are means and standard error of three replicate tanks. Values in the same row followed by different superscript letters are significantly different (*p*  < 0.05) according to Tukeys' multiple comparison test.

Abbreviations: FPH, fish protein hydrolysate; SM, soybean meal.

**Table 7:** Serum biochemical, antioxidant, and immunological parameters in turbot before or after *V. harveyi* challenge.

Item	Control	SM	FPH
Before *V. harveyi* challenge			
Alb (g/L)	7.75 ± 0.60	8.80 ± 1.03	9.97 ± 0.98
TP (g/L)	30.23 ± 1.62	26.22 ± 1.61	30.34 ± 1.12
T-SOD (U/mL)	30.71 ± 0.79	32.40 ± 0.04	32.01 ± 1.27
CAT (U/mL)	29.23 ± 2.02^a^	26.19 ± 1.43^a^	13.45 ± 1.52^b^
GSH-PX (U/mL)	56.92 ± 6.99	52.89 ± 5.09	56.89 ± 5.96
MDA (nmol/mL)	13.22 ± 0.83	13.90 ± 0.41	13.28 ± 0.24
MPO (U/L)	303.45 ± 0.16^b^	338.36 ± 6.19^a^	237.03 ± 8.94^c^
GOT (U/L)	16.26 ± 2.15	24.12 ± 0.94	17.63 ± 2.33
GPT (U/L)	9.59 ± 1.02	11.62 ± 0.62	10.29 ± 1.06
LZM (μg/mL)	7.41 ± 0.26	7.08 ± 0.24	7.94 ± 0.18
T-NOS (U/mL)	26.38 ± 1.96	27.99 ± 1.90	31.45 ± 1.12
IgM (μg/ml)	309.27 ± 17.12^b^	305.88 ± 33.24^b^	472.75 ± 46.12^a^
C3 (μg/ml)	888.02 ± 49.84	766.08 ± 106.48	1011.82 ± 159.79
After *V. harveyi* challenge			
Alb (g/L)	7.77 ± 0.35^a^	3.59 ± 0.63^b^	4.85 ± 0.69^ab^
TP (g/L)	19.17 ± 0.57^b^	12.51 ± 0.84^c^	24.57 ± 0.40^a^
T-SOD (U/mL)	24.55 ± 0.63	26.03 ± 1.08	23.64 ± 1.16
CAT (U/mL)	27.42 ± 2.47	17.17 ± 0.59	20.02 ± 2.60
GSH-PX (U/ml)	18.29 ± 1.89	22.41 ± 2.81	15.75 ± 1.22
MDA (nmol/mL)	9.88 ± 0.27^a^	7.96 ± 0.35^b^	8.59 ± 0.35^ab^
MPO (U/L)	358.68 ± 8.51	375.67 ± 12.42	347.97 ± 15.64
GOT (U/L)	21.98 ± 0.64	20.73 ± 0.95	19.66 ± 2.21
GPT (U/L)	16.51 ± 0.67	14.45 ± 0.41	16.29 ± 0.73
LZM (μg/mL)	7.43 ± 0.10^b^	8.18 ± 0.02^a^	8.14 ± 0.05^a^
T-NOS (U/mL)	17.52 ± 1.21	20.65 ± 0.50	21.47 ± 0.91
IgM (μg/mL)	272.11 ± 17.07	297.39 ± 17.88	321.85 ± 29.06
C3 (μg/mL)	857.06 ± 88.34	927.92 ± 58.80	708.23 ± 45.96

*Note:* Values are means and standard error of three replicate tanks. Values in the same row followed by different superscript letters are significantly different (*p*  < 0.05) according to Tukeys' multiple comparison test.

Abbreviations: ALB, albumin; C3, complement 3; CAT, catalase; FPH, fish protein hydrolysate; GOP, glutamic oxalic aminotransferase; GPT, glutamic pyruvic aminotransferase; GSH-PX, glutathione peroxidase; IgM, immunoglobulin M; LZM, lysozyme; MDA, malondialdehyde; MPO, myeloperoxidase; SM, soybean meal; T-NOS, total nitric oxide synthase; TP, total protein; T-SOD, total superoxide dismutase.

**Table 8:** *Ct* values of the *vhhp*2 gene provided by quantitative real-time-polymerase chain reaction (qRT-PCR) in the intestine after *V. harveyi* challenge.

Item	Control	SM	FPH
Middle intestine	27.29 ± 1.55	30.99 ± 1.19	30.66 ± 0.61
Distal intestine	26.26 ± 1.61^b^	30.59 ± 1.14^ab^	31.60 ± 0.69^a^

*Note:* Values are means and standard error of three replicate tanks. Values in the same row followed by different superscript letters are significantly different (*p*  < 0.05) according to Tukeys' multiple comparison test.

Abbreviations: FPH, fish protein hydrolysate; SM, soybean meal.

**Table 9:** Alpha-diversity index of the distal intestinal microbiota of turbot before or after *V. harveyi* challenge.

Item	Sobs	Shannon	Simpson	Ace	Chao1	Pielou-e
Before *V. harveyi* challenge						
Phylum level						
Control	9.00 ± 1.00	0.53 ± 0.07	0.69 ± 0.06	9.35 ± 1.12	9.00 ± 1.00	9.00 ± 1.00
SM	10.00 ± 1.00	0.65 ± 0.25	0.62 ± 0.16	10.33 ± 1.03	10.00 ± 1.00	10.00 ± 1.00
FPH	9.00 ± 2.65	0.19 ± 0.04	0.92 ± 0.03	13.54 ± 0.26	9.33 ± 2.73	9.00 ± 2.65
Genus level						
Control	40.00 ± 14.18	0.73 ± 0.12	0.65 ± 0.06	59.18 ± 26.66	53.32 ± 20.08	0.20 ± 0.02
SM	81.67 ± 14.84	1.16 ± 0.36	0.52 ± 0.17	98.17 ± 10.87	111.83 ± 5.89	0.27 ± 0.08
FPH	52.67 ± 8.76	0.75 ± 0.27	0.63 ± 0.16	61.58 ± 10.09	60.22 ± 10.58	0.19 ± 0.07
After *V. harveyi* challenge						
Phylum level						
Control	10.00 ± 2.08	0.63 ± 0.12	0.66 ± 0.07	12.91 ± 2.65	11.00 ± 2.08	0.27 ± 0.04
SM	10.00 ± 1.73	0.62 ± 0.18	0.65 ± 0.12	11.44 ± 1.40	10.28 ± 1.59	0.27 ± 0.07
FPH	8.67 ± 1.45	0.34 ± 0.18	0.81 ± 0.14	10.37 ± 1.37	9.17 ± 1.74	0.16 ± 0.08
Genus level						
Control	67.67 ± 22.42	0.89 ± 0.24	0.60 ± 0.10	85.71 ± 32.93	86.80 ± 34.36	0.21 ± 0.04
SM	73.67 ± 18.98	1.10 ± 0.31	0.56 ± 0.14	107.47 ± 22.83	101.75 ± 24.77	0.26 ± 0.07
FPH	51.33 ± 6.89	0.56 ± 0.24	0.76 ± 0.15	79.04 ± 2.38	81.43 ± 13.87	0.14 ± 0.06

*Note:* Values are means and standard error of three replicate tanks.

Abbreviations: FPH, fish protein hydrolysate; Pielou_e, pielou's evenness; SM, soybean meal.

## Data Availability

The datasets of microbial communities have been submitted to online repositories (https://www.ncbi.nlm.nih.gov/, PRJNA1111883). Other data can be made available on request.

## Supporting Information

Supporting Information 1 The supporting information includes Tables S1 and S2 and Figures S1−S6. Table S1: average relative abundance at the phylum and genus levels of the distal intestinal microbiota in turbot before *V. harveyi* challenge; Table S2: average relative abundance at the phylum and genus levels of the distal intestinal microbiota in turbot after *V. harveyi* challenge.

Supporting Information 2 Figure S1: Serum biochemical, antioxidant, and immunological parameters of all treatments were compared before and after *V. harveyi* challenge; Figure S2: The gene expression of immune-related inflammatory cytokines in the proximate intestine was compared before and after *V. harveyi* challenge; Figure S3: The gene expression of immune-related inflammatory cytokines in the middle intestine were compared before and after *V. harveyi* challenge; Figure S4: The gene expression of immune-related inflammatory cytokines in the head kidney were compared before and after *V. harveyi* challenge; Figure S5: Alpha-diversity index of the distal intestinal microbiota at phylum level were compared before and after *V. harveyi* challenge; Figure S6: Alpha-diversity index of the distal intestinal microbiota at genus level were compared before and after *V. harveyi* challenge.
