# Supplementary material for: Exploring Antibacterial Activity of Fish Protein Hydrolysate In Vitro Against Vibrio Strains and Disease Resistance to V. harveyi in Turbot (Scophthalmus maximus)
Source: Aquac Nutr. 2025 Jan 28;2025:3446155. doi: 10.1155/anu/3446155 (PMC11824708; doi:10.1155/anu/3446155)
Supplement: Supporting Information 1 — The supporting information includes Tables S1 and S2 and Figures S1−S6. Table S1: average relative abundance at the phylum and genus levels of the distal intestinal microbiota in turbot before V. harveyi challenge; Table S2: average relative abundance at the phylum and genus levels of the distal intestinal microbiota in turbot after V. harveyi challenge. [file 3446155.f1.docx]

**Table captions**

Table S1 Average relative abundance at the phylum and genus levels of the distal intestinal microbiota in turbot before *V. harveyi* challenge

Table S2 Average relative abundance at the phylum and genus levels of the distal intestinal microbiota in turbot after *V. harveyi* challenge

Table S1 Average relative abundance at the phylum and genus levels of the distal intestinal microbiota in turbot before *V. harveyi* challenge (%)

|  | CON | SM | FPH |
| --- | --- | --- | --- |
| Phylum level |  |  |  |
| *Proteobacteria* | 85.72 | 73.32 | 97.5 |
| *Firmicutes* | 12.63 | 20.15 | 1.13 |
| *Actinobacteria* | 1.13 | 5.18 | 0.51 |
| *Bacteroidota* | 0.18 | 0.19 | 0.27 |
| *Verrucomicrobia* | 0.04 | 0.43 | 0 |
| *Chloroflexi* | 0.08 | 0.28 | 0.03 |
| *Planctomycetota* | 0.01 | 0.17 | 0.12 |
| *Gemmatimonadetes* | 0.09 | 0.03 | 0.11 |
| Others | 0.12 | 0.25 | 0.33 |
| Genus level |  |  |  |
| *Achromobacter* | 83.19 | 69.22 | 81.64 |
| *Bacillus* | 11.83 | 16.63 | 0.44 |
| *Photobacterium* | 0 | 1.01 | 13.06 |
| *Acidimicrobiales* | 0.58 | 2.75 | 0.06 |
| *Acinetobacter* | 0.98 | 0.43 | 1.77 |
| *Staphylococcus* | 0.30 | 1.96 | 0.31 |
| *Pseudomonas* | 0.47 | 0.36 | 0.20 |
| *Georgenia* | 0.02 | 0.85 | 0 |
| *Leifsonia* | 0.10 | 0.35 | 0.30 |
| *Novibacillus* | 0 | 0.46 | 0 |
| Others | 2.53 | 5.98 | 2.22 |

Table S2 Average relative abundance at the phylum and genus levels of the distal intestinal microbiota in turbot after *V. harveyi* challenge (%)

|  | CON | SM | FPH |
| --- | --- | --- | --- |
| Phylum level |  |  |  |
| *Proteobacteria* | 81.39 | 73.28 | 97.25 |
| *Firmicutes* | 16.72 | 23.25 | 1.24 |
| *Actinobacteria* | 1.46 | 2.80 | 1.07 |
| *Bacteroidota* | 0.09 | 0.02 | 0.19 |
| *Chloroflexi* | 0.01 | 0.21 | 0.04 |
| *Gemmatimonadetes* | 0 | 0.18 | 0.08 |
| *Planctomycetota* | 0.13 | 0 | 0.06 |
| *Verrucomicrobia* | 0.17 | 0.01 | 0 |
| Others | 0.03 | 0.25 | 0.07 |
| Genus level |  |  |  |
| *Achromobacter* | 80.1 | 71.36 | 95.5 |
| *Bacillus* | 16.33 | 0.52 | 0.62 |
| *Clostridium* | 0.01 | 14.42 | 0.06 |
| *Vibrio* | 0.20 | 0.92 | 0.50 |
| *Acidimicrobiales* | 0.24 | 0.65 | 0.45 |
| *Micrococcus* | 0.45 | 0.17 | 0.16 |
| *Pseudomonas* | 0.21 | 0.15 | 0.33 |
| *Leifsonia* | 0.16 | 0.25 | 0.27 |
| *Acinetobacter* | 0.10 | 0.54 | 0.02 |
| *Corynebacterium* | 0.25 | 0.25 | 0.10 |
| Others | 1.95 | 10.77 | 1.99 |
