# Supplementary material for: Exploring Antibacterial Activity of Fish Protein Hydrolysate In Vitro Against Vibrio Strains and Disease Resistance to V. harveyi in Turbot (Scophthalmus maximus)
Source: Aquac Nutr. 2025 Jan 28;2025:3446155. doi: 10.1155/anu/3446155 (PMC11824708; doi:10.1155/anu/3446155)
Supplement: Supporting Information 2 — Figure S1: Serum biochemical, antioxidant, and immunological parameters of all treatments were compared before and after V. harveyi challenge; Figure S2: The gene expression of immune-related inflammatory cytokines in the proximate intestine was compared before and after V. harveyi challenge; Figure S3: The gene expression of immune-related inflammatory cytokines in the middle intestine were compared before and after V. harveyi challenge; Figure S4: The gene expression of immune-related inflammatory cytokines in the head kidney were compared before and after V. harveyi challenge; Figure S5: Alpha-diversity index of the distal intestinal microbiota at phylum level were compared before and after V. harveyi challenge; Figure S6: Alpha-diversity index of the distal intestinal microbiota at genus level were compared before and after V. harveyi challenge. [file 3446155.f2.docx]

**Figure captions**

Figure S1 Serum biochemical, antioxidant and immunological parameters of all treatments were compared before and after *V. harveyi* challenge

Figure S2 The gene expression of immune-related inflammatory cytokines in the proximate intestine were compared before and after *V. harveyi* challenge

Figure S3 The gene expression of immune-related inflammatory cytokines in the middle intestine were compared before and after *V. harveyi* challenge

Figure S4 The gene expression of immune-related inflammatory cytokines in the head kidney were compared before and after *V. harveyi* challenge

Figure S5 Alpha-diversity index of the distal intestinal microbiota at phylum level were compared before and after *V. harveyi* challenge

Figure S6 Alpha-diversity index of the distal intestinal microbiota at genus level were compared before and after *V. harveyi* challenge


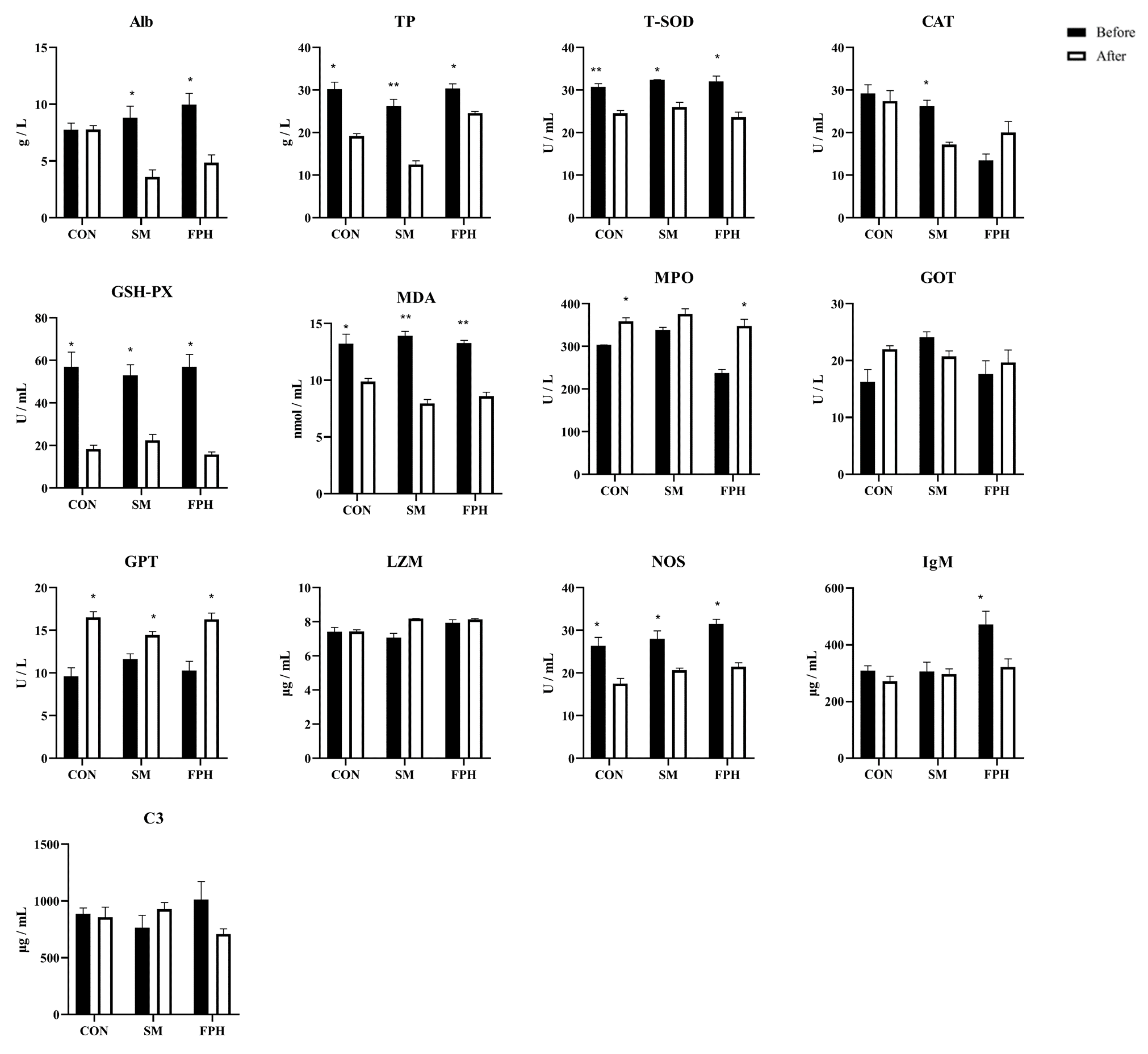


Figure S1 Serum biochemical, antioxidant and immunological parameters of all treatments were compared before and after *V. harveyi* challenge. Alb: albumin; TP: total protein; T-SOD: total superoxide dismutase; CAT: catalase; GSH-PX: glutathione peroxidase; MDA: malondialdehyde; MPO: myeloperoxidase; GOP: glutamic oxalic aminotransferase; GPT: glutamic pyruvic aminotransferase; LZM: lysozyme; T-NOS: total nitric oxide synthase; IgM: immunoglobulin M; C3: Complement 3. Values are means ± standard error of three replicate tanks. Significant difference was performed according to independent sample T-test and indicated by asterisk, **P* < 0.05, ***P* < 0.01.


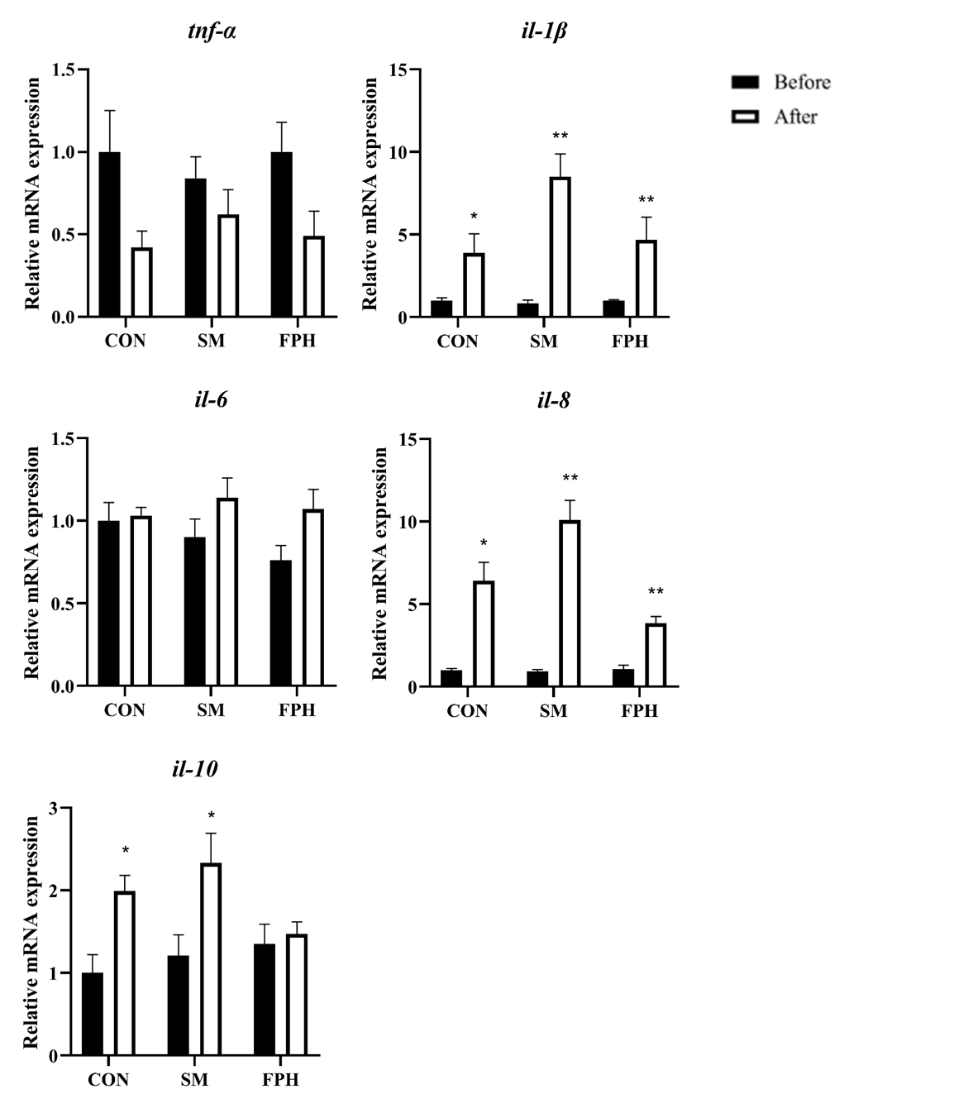


Figure S2 The gene expression of immune-related inflammatory cytokines in the proximate intestine were compared before and after *V. harveyi* challenge. *tnf-α*: tumor necrosis factor *α*; *il-1β*: interleukin-1*β*. Values are means ± standard error of three replicate tanks. Significant difference was performed according to independent sample T-test and indicated by asterisk, **P* < 0.05, ***P* < 0.01.


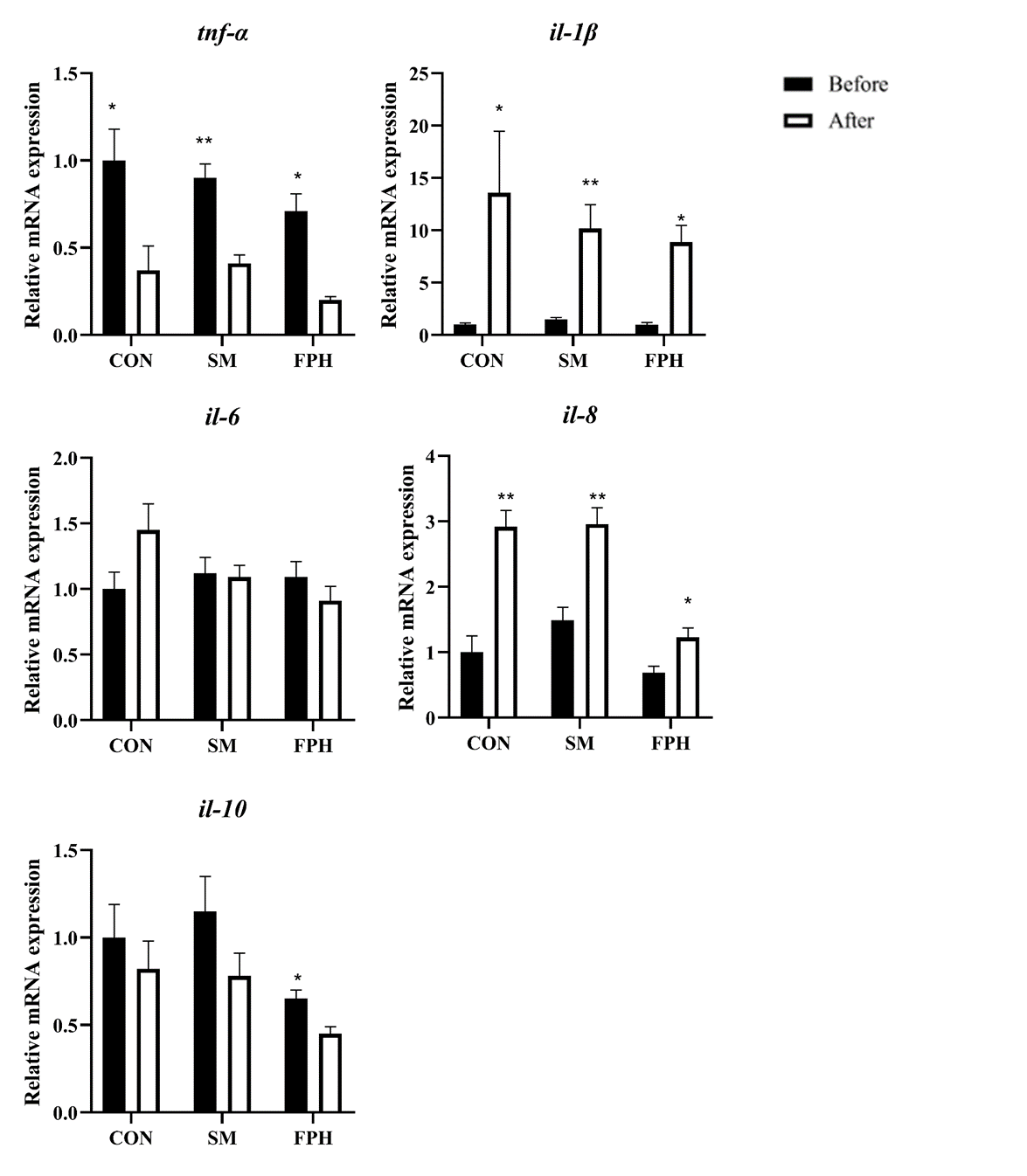


Figure S3 The gene expression of immune-related inflammatory cytokines in the middle intestine were compared before and after *V. harveyi* challenge. *tnf-α*: tumor necrosis factor *α*; *il-1β*: interleukin-1*β*. Values are means ± standard error of three replicate tanks. Significant difference was performed according to independent sample T-test and indicated by asterisk, **P* < 0.05, ***P* < 0.01.


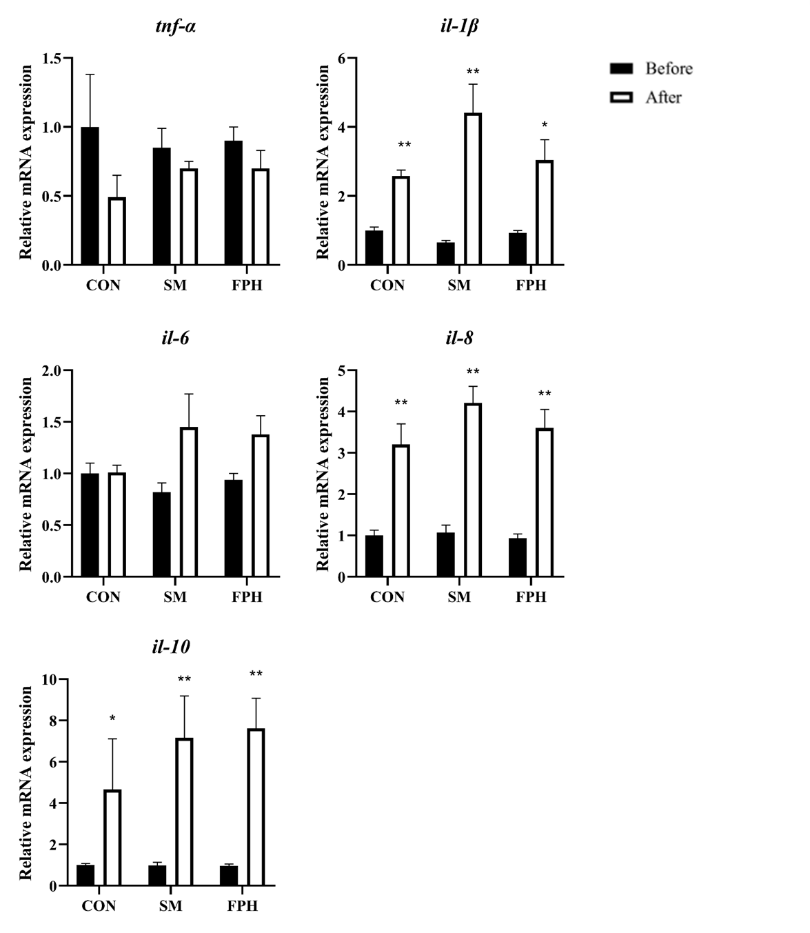


Figure S4 The gene expression of immune-related inflammatory cytokines in the head kidney were compared before and after *V. harveyi* challenge. *tnf-α*: tumor necrosis factor *α*; *il-1β*: interleukin-1*β*. Values are means ± standard error of three replicate tanks. Significant difference was performed according to independent sample T-test and indicated by asterisk, **P* < 0.05, ***P* < 0.01.


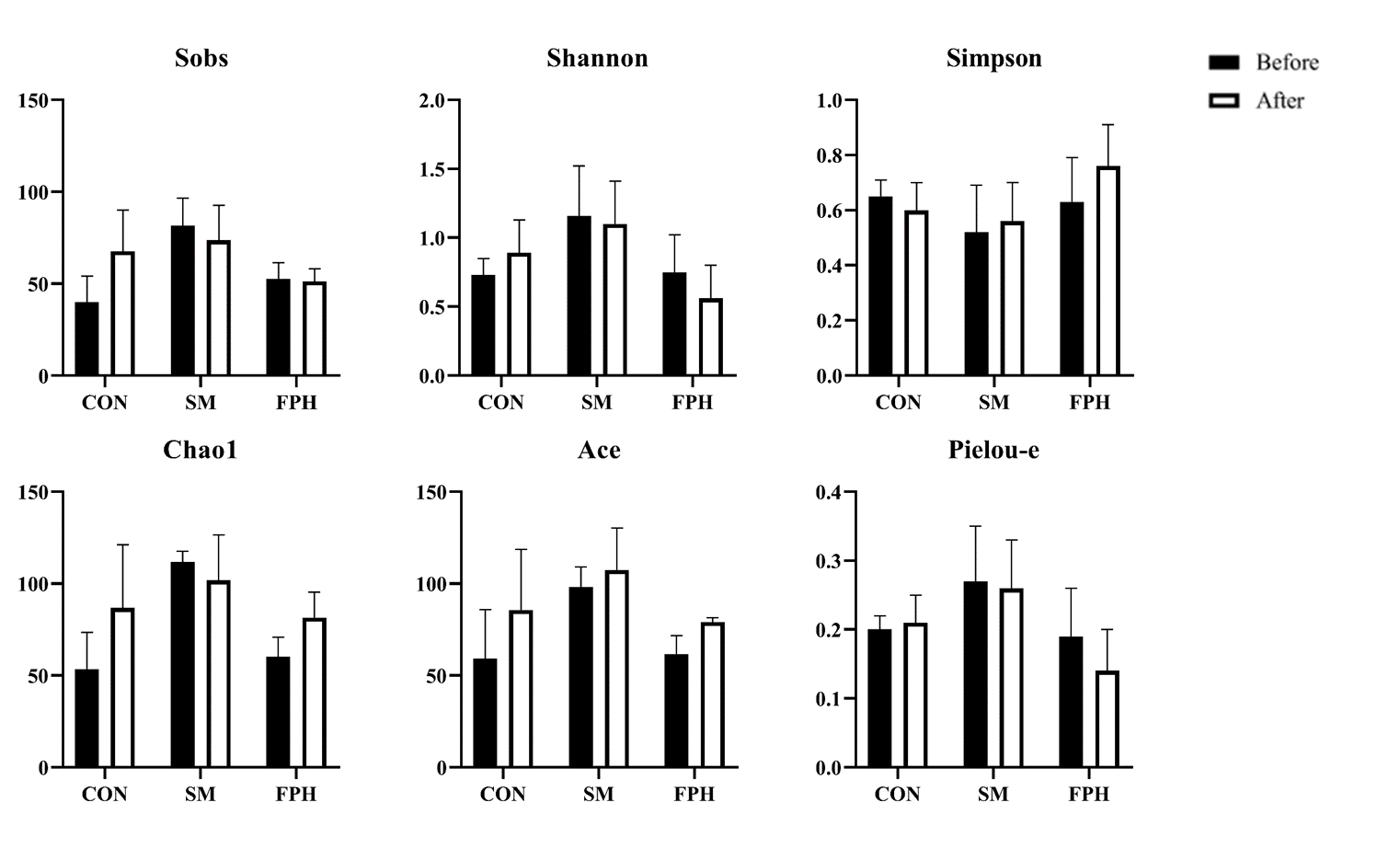


Figure S5 Alpha-diversity index of the distal intestinal microbiota at phylum level were compared before and after *V. harveyi* challenge. Values are means ± standard error of three replicate tanks. Significant difference was performed according to independent sample T-test.


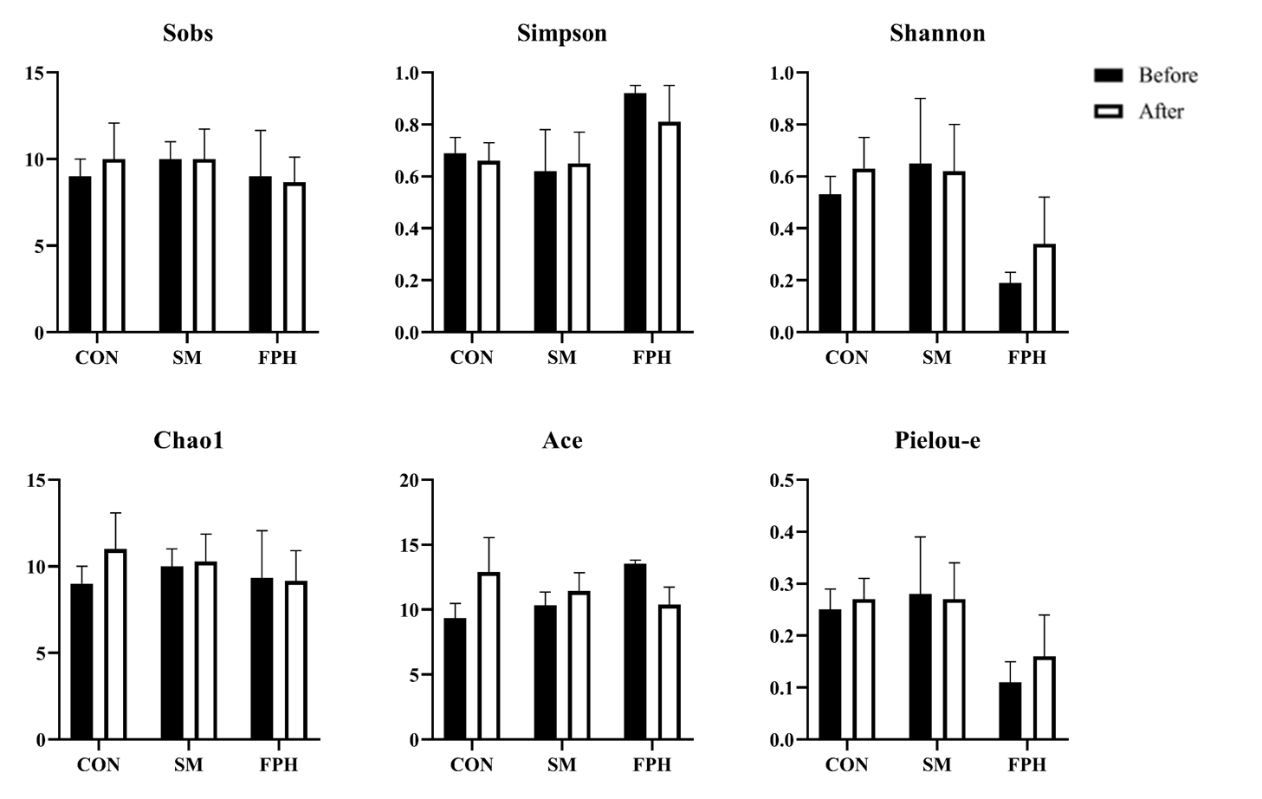


Figure S6 Alpha-diversity index of the distal intestinal microbiota at genus level were compared before and after *V. harveyi* challenge. Values are means ± standard error of three replicate tanks. Significant difference was performed according to independent sample T-test.
